# Disparities in Patient Portal Activation and Usage at a Large Pediatric Academic Institution

**DOI:** 10.1007/s40615-024-02009-w

**Published:** 2024-11-04

**Authors:** Ethan G. Chuang, Andrew C. Richardson, Zaineb Boulil, Cynthia L. Kuelbs, Jeannie S. Huang

**Affiliations:** 1 Department of Pediatrics, University of California San Diego, San Diego, CA USA; 2 Rady Children’s Hospital, San Diego, CA USA

**Keywords:** Electronic medical record, Disparity, Ethnicity, Language

## Abstract

**Background and Objective:**

Access to personal medical information promotes patient understanding of health issues and enables patient self-advocacy of healthcare needs. The advent of electronic medical record systems and the 2016 21st Century CURES Act promoted and encouraged patient access to personal medical information, yet technology-dependent modalities have often disadvantaged certain communities. We sought to evaluate whether disparities existed in access to patient portals at our institution, the main pediatric care provider in an area serving one million children.

**Methods:**

We evaluated the activation of patient portal accounts at our institution over the past decade (2010–2021). Portal activation data were analyzed by ethnic background and language preference and income information available based on primary home access location. Further, we evaluated portal activation rates over time and across institutional interventions. A logistic regression model was used to identify important demographic associations with portal account status.

**Results:**

Over 1 million patients were served at our pediatric institution over the study period with 47.7% having ever activated their patient portals. Univariate analyses and ultimately logistic regression modeling demonstrated significant differences in portal activation by ethnicity (odds ratio (confidence interval):1.36 (1.34, 1.37)), language preference (1.39 (1.37, 1.40)), and median household income (1.00001 (1.00001, 1.00001)). Interim interventions were successful in improving portal activation rates.

**Discussion:**

Overall, electronic medical record portal activation was less prevalent among Hispanic, non-English speakers, and patients living in communities with lower median household income.

**Conclusion:**

Opportunities for interventions exist to reduce healthcare disparities in these underserved communities.

## Background and Significance

The WHO’s Global Strategy on Digital Health 2020–2024 promotes adoption of an increased use of digital health technologies to improve healthcare access and delivery “to promote healthy lives and wellbeing for everyone, everywhere, at all ages [1].” With the advent and almost universal adoption of electronic medical records (EMR) [2], EMR-associated patient portals have become the primary method for patient-physician communications. The 21st Century Cures Act [3] has enabled unprecedented patient access to electronic personal health and healthcare information via EMR-enabled patient portals. Further, with the recent COVID-19 pandemic, there has been an increase in the activation of patient portals due to promotion of and increased availability of telehealth to reduce pandemic spread [4]. Patient portals allow patients to access a wide variety of personal medical information, including details of recent appointments, medications, immunizations, prescriptions, and lab results. Use of patient portals among adults related to their own health and healthcare has been associated with additional health benefits, such as improved ambulatory care engagement and an increase in adult patients’ self-care and adherence to medical treatment, improved relationships with their physicians, shared decision-making, and improved chronic disease outcomes [5, 6].

While such digital health expansions have been beneficial, already existing healthcare disparities have been exacerbated. Prior work in adults has shown that patients with certain demographics such as male, non-White, having Medicaid insurance, lower income, and education achievement, lacking a regular provider, and having poor internet access were found to be less likely to activate their online portal account as compared to counterparts [7]. In addition, Hispanic adults and adults preferring a non-English language are significantly less likely to be portal adopters [8]. Finally, an evaluation using data from the 2019 Health Information National Trends Survey (HINTS) which collects data on individuals’ use of and access to health-related information, and health-related knowledge, awareness, and behaviors from civilian noninstitutionalized adults living in the United States identified the most common barriers to patient portal activation: preference for in-person communication, not having a need for the patient portal, and feeling uncomfortable with computers [9].

## Objective

EMR portal access data are limited in pediatric populations. We evaluated patient portal data at our pediatric healthcare institution to understand patient portal activation and usage patterns in pediatric health systems. Given documented disparities in portal activation among adult patients based on demographic factors [7, 8], we sought to evaluate whether similar patient portal account activation disparities were present at our pediatric academic healthcare institution. By understanding existing patterns, and patterns over time with portal account targeted interventions, we hoped to identify disparities in access to pediatric health information to inform future interventions targeting pediatric patient portal activation and usage.

## Materials and Methods

We are a free-standing children’s hospital in a large metropolitan region serving a catchment population of > 1 million children. Patient portal account access was enabled in 2010 with implementation of our EMR system (Epic Systems, Verona, WI). For adolescent patients 12–18 years, teen setup of separate portal accounts is encouraged, and parent permission is required for activation of teen accounts. Our study was determined to be exempted from institutional review because it was a quality improvement project. For the purposes of this analysis, we determined whether patient health information was accessible through any portal account. Portal account activation was not subcategorized by teen v. proxy account status.

Primary patient population criteria included patients who had a clinical encounter between 01/01/2010 through 12/31/2021 and who were < 18 years at the time of the encounter. Eligible encounter types included inpatient, emergency, urgent care, and ambulatory appointments at hospital-based specialty clinics.

EMR portal activation status and usage data were collected across 2010–2021. Patient portal account status was recorded as activated, pending activation (initiated but not yet completed), inactivated (due to failed logins, deactivated by administration, or because portal terms and conditions declined), and never activated. Never activated status was further subcategorized by reasons why activation processes were not initiated: never offered and patient declined. 

Demographic data were collected. Ethnicity data was classified as Hispanic v. non-Hispanic v. Patient Refused v. Unknown. Preferred language data was classified as English, Spanish, or Other with the last catch-all group containing any language not English or Spanish. Owing to the small numbers represented in missing or unavailable ethnic data groupings and in the other language group, comparisons by ethnicity were between Hispanic v. non-Hispanic and between Spanish v. English, respectively.

### Portal Activation Interventions

Two institutional interventions were performed to improve portal activation rates over the evaluation period. When we first launched our electronic medical record system, portal activation was encouraged but not performed using standard protocols. In 2013, system protocols were established directing registration staff to sign-up all patients without a portal account at the time of check-in for all clinical encounters. By instituting this change, the onus of portal activation was redirected from the patient to the system.

Another systemwide intervention was performed in 2020 in response to the COVID-19 pandemic to facilitate telehealth visits where off-site parent/caregiver portal registration was enabled. Prior to this, all portal activations were performed only in person. During the COVID-19 pandemic, processes were put in place to enable parents and caregivers to sign up and activate their portal account over the telephone.

Throughout the evaluation period, monthly portal activation rates have been reported by clinic to provide feedback to staff and to continue to encourage portal activation.

### Statistical Analyses

Chi-squared analyses were used to evaluate the relationships between language and ethnicity and portal account status. Violin plots were used to visualize population income data and portal account status. Mean/median comparisons were performed using ANOVA analyses for parametric variables and Kruskal–Wallis for nonparametric variables.

Logistic regression modeling was used to determine the relative contribution of identified significant univariate relationships.

Over the study period, two institutional interventions were performed to improve portal account activation rates. Analysis of the efficacy of these interventions were performed via nonparametric repeated measures analysis.

## Results

### Study Cohort

Over one million (1,053,254) patients received healthcare at our institution over an 11-year period (2010–2021). Demographic characteristics are listed in Table 1. 39.6% were Hispanic and 31% white non-Hispanic. 82.2% identified English as their preferred language, 13.3% Spanish, and 4.5% Other. The mean and median current age of the served patient population was 13.0 years and 12.6 years, respectively. 47.8% of the patient population was female and 52.2% male.

**Table 1:** Patient cohort demographics (*N* = 1,053,254)

Demographic	Results *N* (%)
Sex	503,385 (47.8%) F 549,402 (52.2%) M 477 (< 1%) unknown
Race	American Indian 2837 (0.3%) Asian 56,548 (5.4%) Black/African American 47,977 (4.6%) Hispanic 183,832 (17.4%) Middle Eastern/North African 2765 (0.3%) Multiracial 45,533 (4.3%) Native Hawaiian 759 (0.1%) Other 289,833 (27.5%) Other Pacific Islander 6837 (0.6%) White Non-Hispanic 326,168 (31.0%) Decline to answer 16,139 (1.5%) Unknown 74,026 (7.0%)
Ethnicity	Hispanic/Latino 416,909 (39.6%) Non-Hispanic 563,231 (53.5%) Patient refused 10,771 (1.0%) Unknown 62,343 (5.9%)
Preferred language	English 865,684 (82.2%) Spanish 140,459 (13.3%) Other 47,111 (4.5%)

### EMR Patient Portal Status by Ethnicity and Preferred Language

**Fig. 1:**
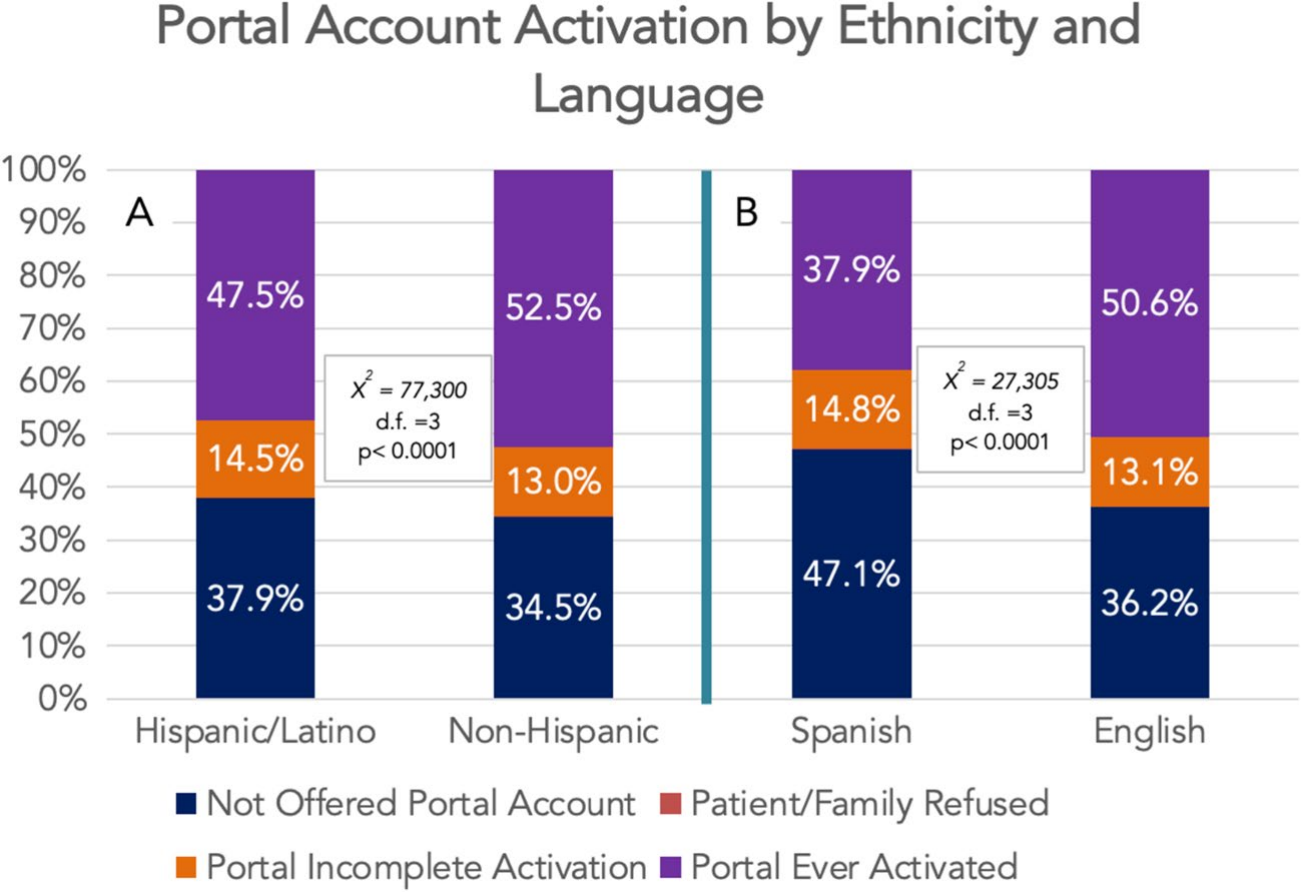
Portal activation status by **A** ethnicity and **B** language

Most of the served patient population had activated their portal account (activated + inactivated = 47.7%) or were in the process of portal account activation (13.1%). Portal activation statuses by ethnicity and preferred language are displayed in Fig. 1. Significant portal account activation disparities were observed by both ethnicity and preferred language with Hispanic and Spanish-preferred language patients demonstrating lower portal activation rates (Hispanic v. non-Hispanic, 47.5% v. 52.5% activation rates, *p* < 0.0001; and Spanish v. English, 37.9% v. 50.6% activation rates, *p* < 0.0001). Further, increased rates of lack of offering portal account activation were seen among Hispanic (Hispanic v. non-Hispanic, 37.9% v. 34.5% lack of offering portal account, *p* < 0.0001) and Spanish-speaking patients (Spanish v. English, 47.1% v. 36.2%, *p* < 0.0001) compared to relative counterparts.

### Income Data

**Fig. 2:**
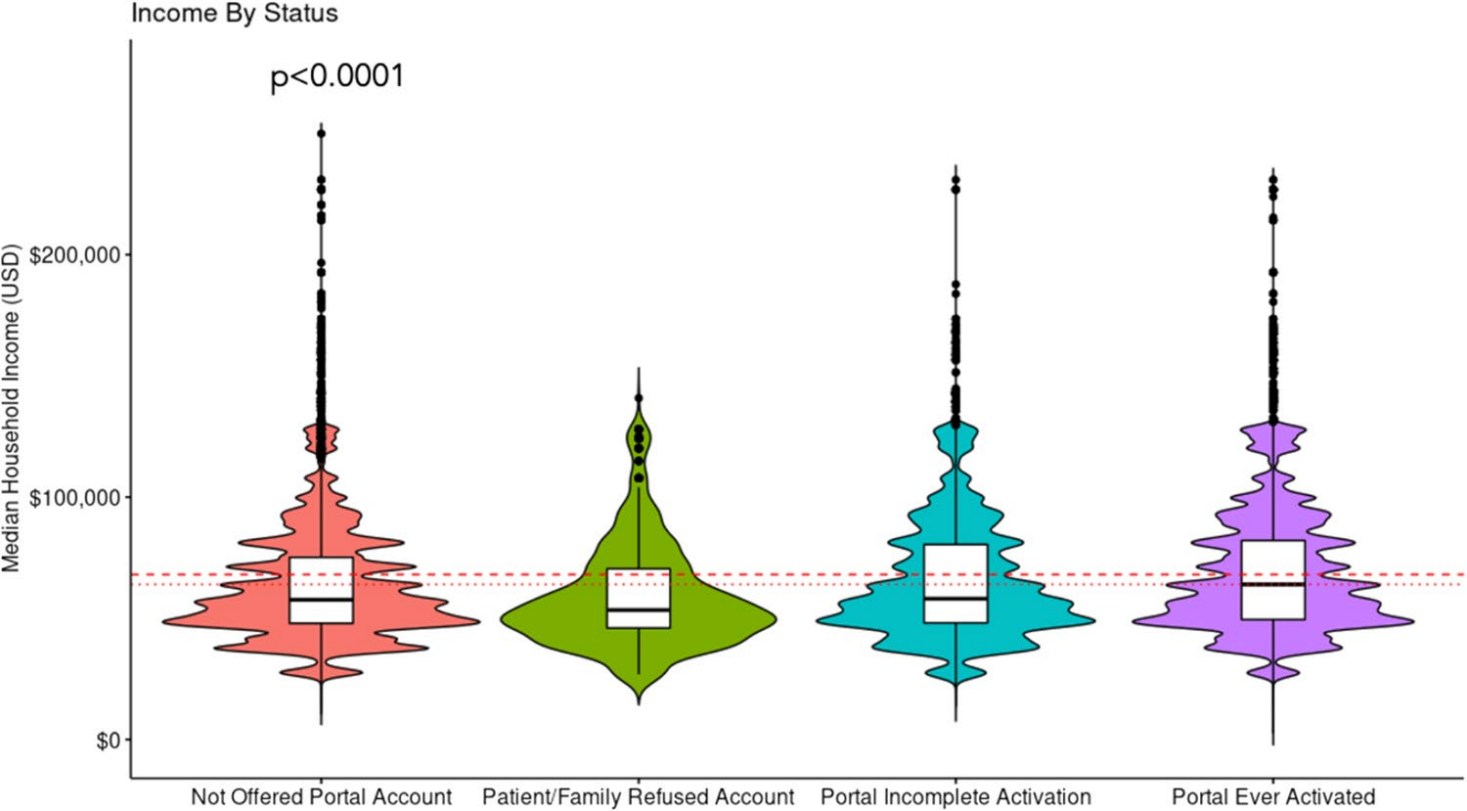
Income by portal activation status

Median income level significantly varied by portal account activation status (*p* < 0.0001, Fig. 2). Median income level was highest among patients who activated their portal account (median (IQR): $64,004 ($32,554)) and was the lowest among patients who refused a portal account ($53,448 ($24,478)).

### Multivariate Model

Multivariate modeling to predict whether a portal account had ever been activated demonstrated significant associations with preferred language, ethnicity, and income (Table 2). Specifically, English language preference, non-Hispanic ethnicity, and higher median income were associated with greater likelihood of portal activation than relevant counterparts.

**Table 2:** Logistic regression model predicting whether a portal account had ever been activated

Variable	Odds ratio	Confidence interval	*p*-value
Language (English v. non-English)	1.39	(1.37, 1.40)	< 0.0001
Ethnicity (non-Hispanic v. Hispanic)	1.36	(1.34, 1.37)	< 0.0001
Median household income	1.000006	(1.000006, 1.0000061)	< 0.0001

### Portal Account Activation Interventions and Their Efficacy

**Fig. 3:**
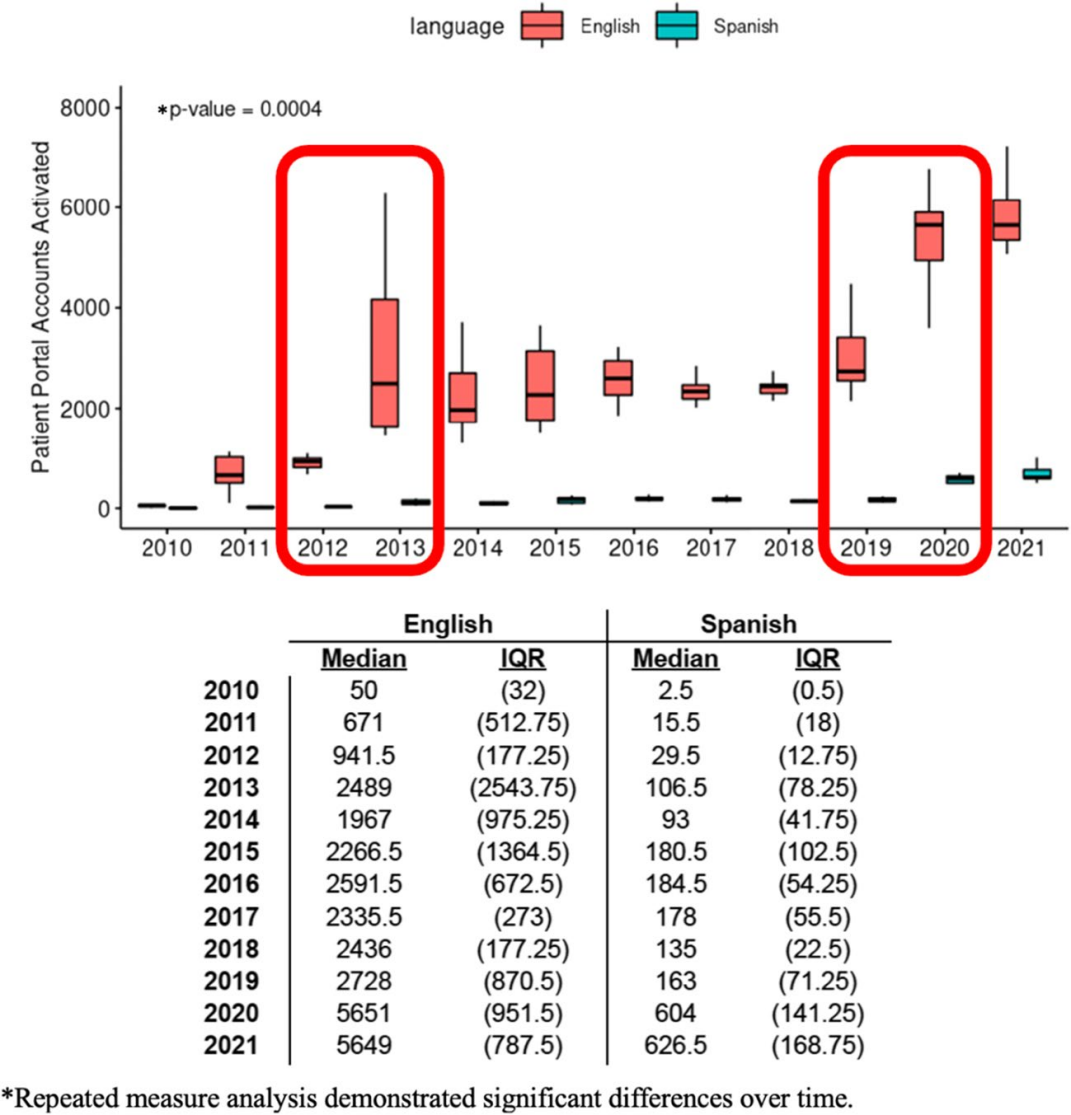
Patient portal activations by year by language. Intervention periods are highlighted by red outlined boxes

As shown in Fig. 3, portal account activation rates overall improved over time (repeated measure analysis *p* = 0.0004) and correlated with graphically visible differences in 2013 and 2020 when system wide interventions were performed. Between these interventions, portal activation rates increased most notably in 2020 across languages with a several-fold increase specifically for preferred Spanish language account activations. Nevertheless, discrepancies remained according to preferred language as shown in Fig. 1.

## Discussion

Over an 11-year period, we demonstrate notable disparities in patient portal activation based on ethnicity and language preference at our institution serving a catchment area of > 1 million children. These disparities persisted despite effective institutional interventions to improve patient portal activation rates. 

Receiving and understanding health information is crucial to patient engagement in their own healthcare, as validated through work by the OpenNotes initiative and others [3, 4, 10]. Already, Hispanic patients and non-English speakers have been shown to have worse health outcomes as compared to white, English-speaking counterparts [11, 12]. Our work demonstrates that Hispanic and Spanish speaking families do not currently have the same access to EMR patient portals. Lack of access to health information to address personal health needs may contribute to already existing health disparities. If we do not intervene, we could potentially further disadvantage and marginalize these at-risk populations.

Both individual and systemic factors contribute to lack of patient portal activation and usage. Identified individual reasons for lack of patient portal activation include preference for in-person communication, not having a need for the patient portal, and feeling uncomfortable with computers [9]. This same research demonstrated that Hispanic individuals were more likely to feel that they had no need for a patient portal compared with non-Hispanic individuals. Further, our recent study evaluating factors for non-adoption of telemedicine for pediatric health concerns demonstrated that Hispanic families preferred in-person to telemedicine visits for discussion of a new medication or surgical procedure and discussing their child’s behavioral or mental health issues [13]. In this study, strong parent-provider relationships positively influenced perceived adequacy of telemedicine and patient willingness to adopt telemedicine. This study highlights the potential role that physicians can play in promoting portal activation and utilization. Having physicians tout the benefits of patient portals would help patients better understand their usefulness and how portals and information accessed through portals can improve patient engagement and outcomes. While individual barriers to portal activation and use are notable, they are modifiable and can be targets for future interventions. Patient portal educational videos have already been shown promise in improving adolescent and young adult understanding of patient portal functions [14].

System-level interventions are also needed to improve patient portal activation. At a pediatric subspecialty clinic, engagement of all members of the clinical team to activate patient portal increased rates by 1.8 to 30% over a 6-month period [15]. At our pediatric institution, through two systemwide interventions, we similarly successfully engaged members of the healthcare team to improve registration rates. While these interventions did improve portal activation rates overall, these interventions were not sufficient to overcome observed disparities in portal account activation. Clearly, more tailored interventions are needed to address the concerns and barriers in the Hispanic, Spanish-speaking, and lower income communities.

Regarding how portal account interventions should be tailored, data from the HINTS survey demonstrate that provider encouragement regarding portal account activation plays a positive role in Black and Hispanic individuals’ activating and using patient portals [16]. Similar to our own findings, HINTS data from 2014 to 2022 demonstrated overall increased access to and engagement with patient portals but persistent racial and ethnic disparities in portal account activations over time [17]. While access to portal accounts differed by race and ethnicity, no significant differences among groups in use or understanding of information presented via portal accounts suggest that the main barrier to health information utility appears to be access to patient portals. Taken together, these findings suggest that portal account interventions should primarily target account activation and capitalize on provider encouragement to motivate account activation by addressing the benefits of portal accounts.

Limitations to our findings include its inclusion of data from only one institution. However, our institution is the primary pediatric care provider in the region and data thus accurately represents local portal account activation and behaviors. Another limitation is that we did not distinguish portal activation and behaviors by teen versus parent proxy account. Portal activation and access behaviors are important to understand in the context of pediatrics as health information sharing in pediatric patient care involves not only the patient but also proxy access by parents. In many systems, including ours, adolescent access to their health information begins at age 12 years to assure confidentiality and privacy related to reproductive health service access. One prior study evaluating EMR access to pediatric data demonstrated differences in access and portal health information access behaviors by age (child v. adolescent v. adult) [18]. We previously demonstrated that health literacy plays an important role in patient portal usage with greater annual patient portal usage (including logins, test result, and medical note viewings) found among adolescents and young adults with chronic disease demonstrating health literacy competency as compared to non-competent counterparts [19]. Unfortunately, a main confounder to data evaluating use of portal accounts by adolescents is the recent discovery of over half of adolescent portal accounts being used by parent proxies [20], a study that included data from our own institution. While subsequent reactive work has been performed to ensure secure access by only portal account owners, the validity of prior data has been brought into question. For this reason, we did not separate out portal activation by teen v. parent proxy account in the current work. Future research is needed to understand portal account access and behaviors in adolescence given the need to promote engagement in their own healthcare and health outcomes and to help prepare adolescents for the transition from pediatric to adult centered care.

## Conclusions

We demonstrate significant differences in patient portal activation rates based on ethnicity and language preference in our 11-year experience at a pediatric healthcare institution. Lower income level also was associated with lower rates of patient portal account activation. Our findings speak to a growing need to better engage Hispanic, non-English speaking, and lower income patient clients regarding portal account activation to address health information sharing disparities.

## Data Availability

The data that support the findings of this study are available from the corresponding author, JSH, upon reasonable request.

## Abbreviations

WHO: World Health Organization
EMR: Electronic medical records
ANOVA: Analysis of variance
